# Correction: Growing evidence of *Plasmodium vivax* across malaria-endemic Africa

**DOI:** 10.1371/journal.pntd.0007525

**Published:** 2019-06-28

**Authors:** The PLOS Neglected Tropical Diseases Staff

## Notice of republication

This article was republished on May 3, 2019, to correct errors in the figures and supporting figures. The publisher apologizes for the errors. Please download this article again to view the correct version. The originally published, uncorrected article and the republished, corrected articles are provided here for reference.

## Supporting information

S1 File Originally published, uncorrected article. (PDF)

S2 File Republished, corrected article. (PDF)
