# Supplementary material for: Growing evidence of Plasmodium vivax across malaria-endemic Africa
Source: PLoS Negl Trop Dis. 2019 Jan 31;13(1):e0007140. doi: 10.1371/journal.pntd.0007140 (PMC6372205; doi:10.1371/journal.pntd.0007140)
Supplement: S2 Checklist — (DOC) [file pntd.0007140.s002.doc]

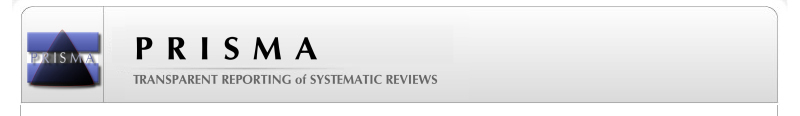
**PRISMA 2009 Flow Diagram**

**Screening**

**Included**

**Eligibility**

**Identification**

Records identified through database searching
(n = 647)

Additional records identified through other sources
(n = 8)

Records after duplicates removed
(n = 388)

Records screened
(n = 375)

Records excluded
(n = 226)

Full-text articles assessed for eligibility
(n = 149)

Full-text articles excluded, with reasons
(n = 53)

Studies included in qualitative synthesis
(n = 96)

Studies included in quantitative synthesis (meta-analysis)
(n = 53)
