# Supplementary figures and images for: Growing evidence of Plasmodium vivax across malaria-endemic Africa
Source: PLoS Negl Trop Dis. 2019 Jan 31;13(1):e0007140. doi: 10.1371/journal.pntd.0007140 (PMC6372205; doi:10.1371/journal.pntd.0007140)

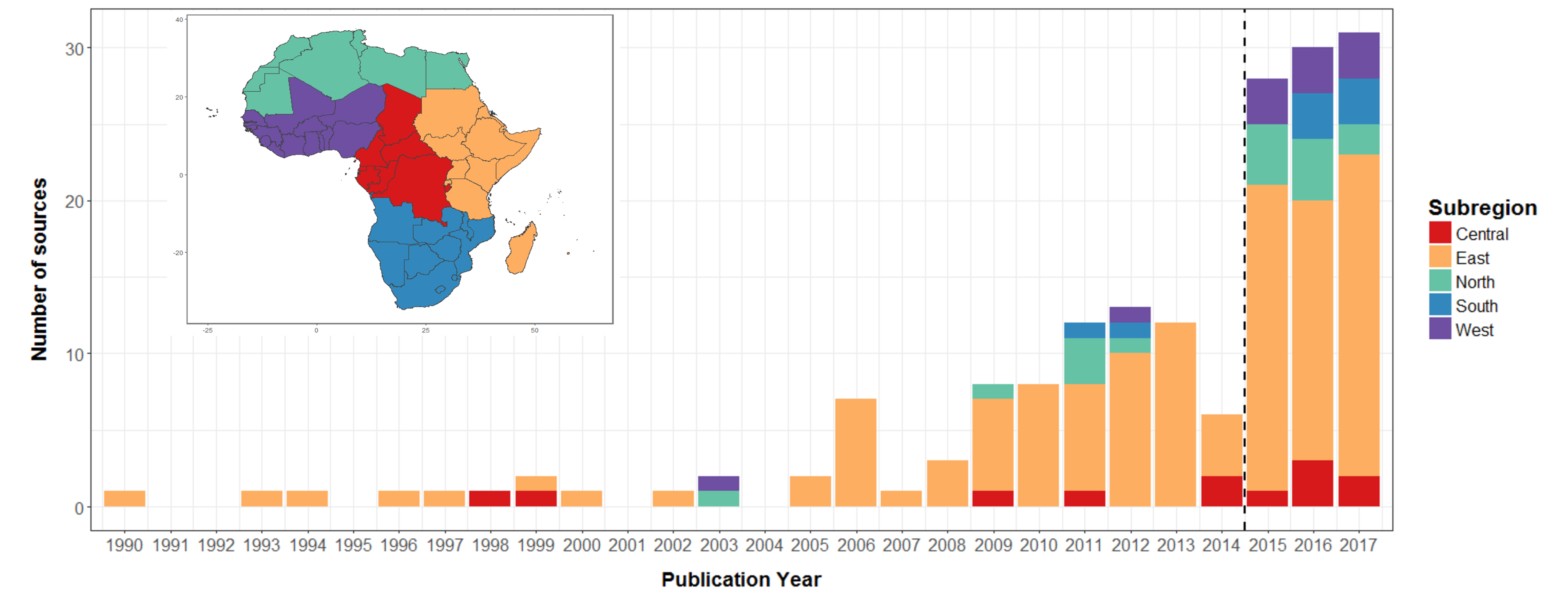

Supplement: S1 Fig — The number of sources (y-axis) by year of publication (x-axis). Bars are coloured according to the African Union region [47] from which data in each source originated (shown in inset map). The dashed line approximately indicates the data sources included in Howes et al. 2015; sources after the line have been added since that publication. Excluded from this plot are nine additional articles from the beginning of 2018 (through to the search date 25/04/2018), and one unpublished report. Occasionally sources would report data from multiple countries and multiple regions, in which case an individual source was counted more than once. However, duplicates were removed to the extent possible to show distinct sources. All unique combinations of source, publication year and region were retained. (TIF) [file pntd.0007140.s003.tif]

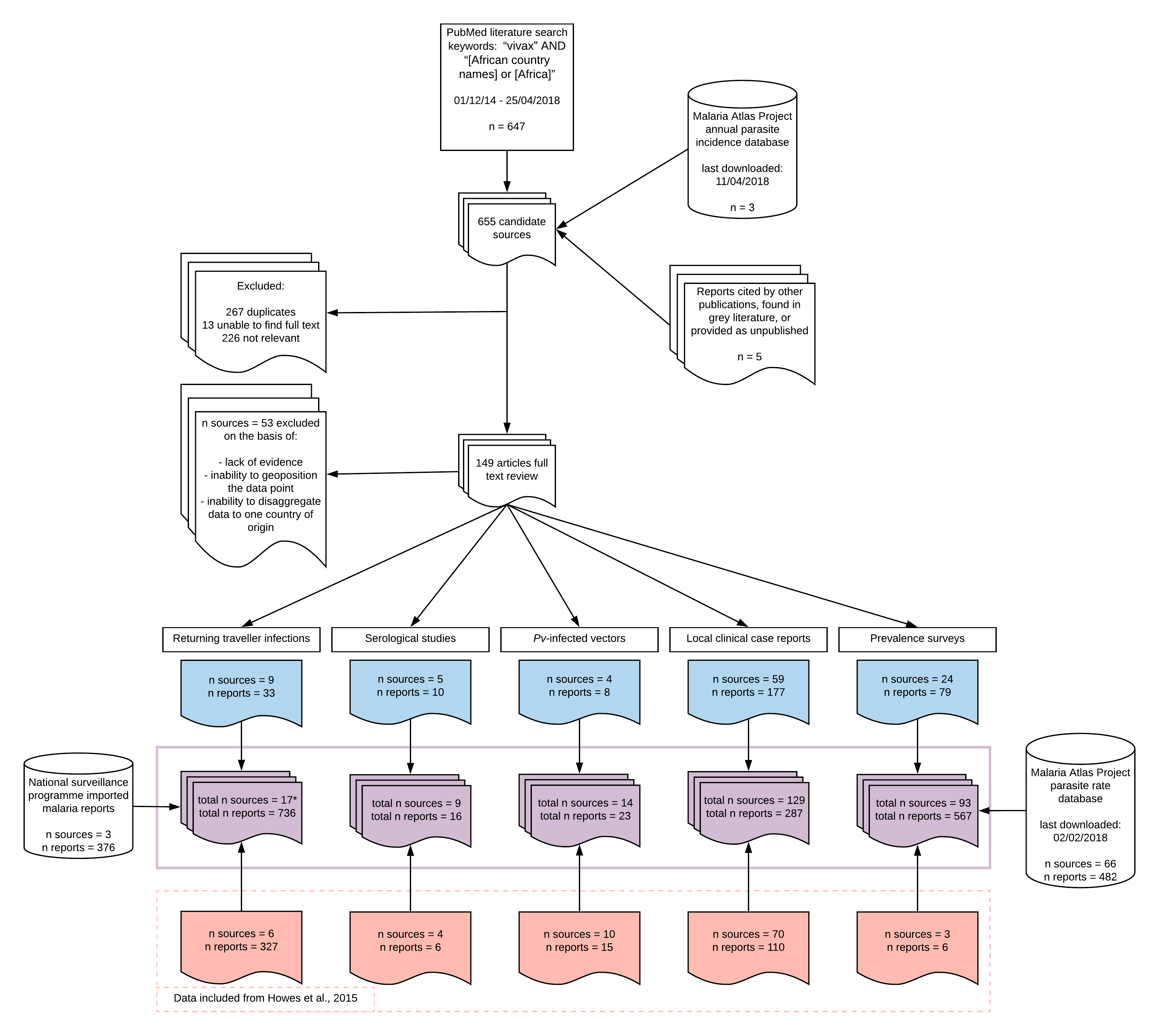

Supplement: S2 Fig — Data was sourced from a comprehensive literature search, the Malaria Atlas Project’s malariometric databases, and contributions of data on imported traveller infections from national/regional infectious disease surveillance programs outside of Africa. Evidence types were categorised, and new data (blue) was combined with data from Howes et al. (red) to yield a final updated evidence base (purple). The number of reports (n reports) refers to the number of spatially and temporally unique points. It is not the same as the number of unique geographic locations. In five instances, evidence was found for multiple data types in the same source. Thus, the number of new unique sources (blue) is 96. The 2018 literature search included all African countries, unlike the previous 2015 search which was limited to countries endemic with malaria in 2014. * The traveller database was updated with additional years of data from the same source in one instance, so the total number of sources is not strictly the sum of all separate contributions. (PNG) [file pntd.0007140.s004.png]

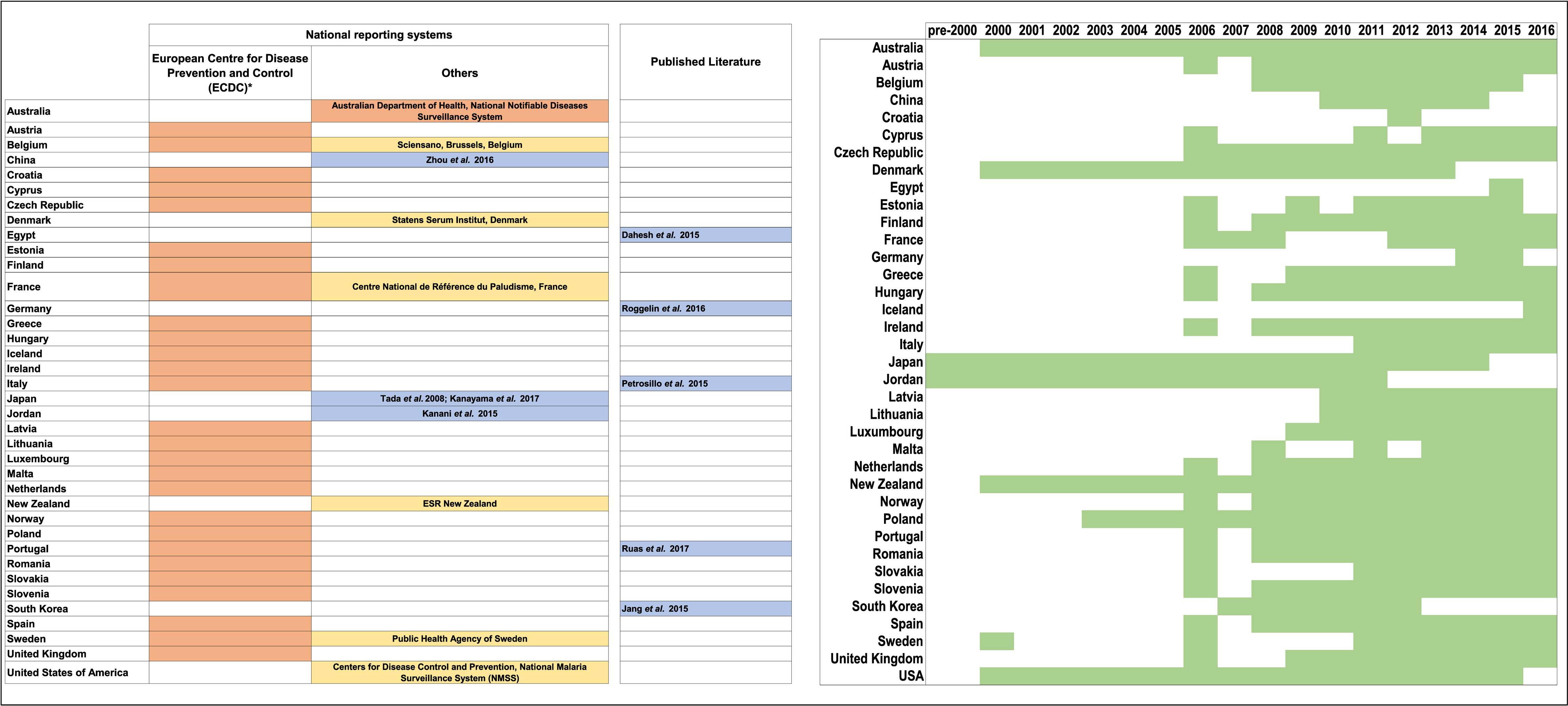

Supplement: S3 Fig — Data on returning traveller infections was mainly collected from national surveillance programmes through data access requests, personal communications, publicly accessible reports and scientific publications of aggregate data. Other data were obtained through the literature search and mainly consisted of case reports not included in the national aggregates. (TIF) [file pntd.0007140.s005.tif]

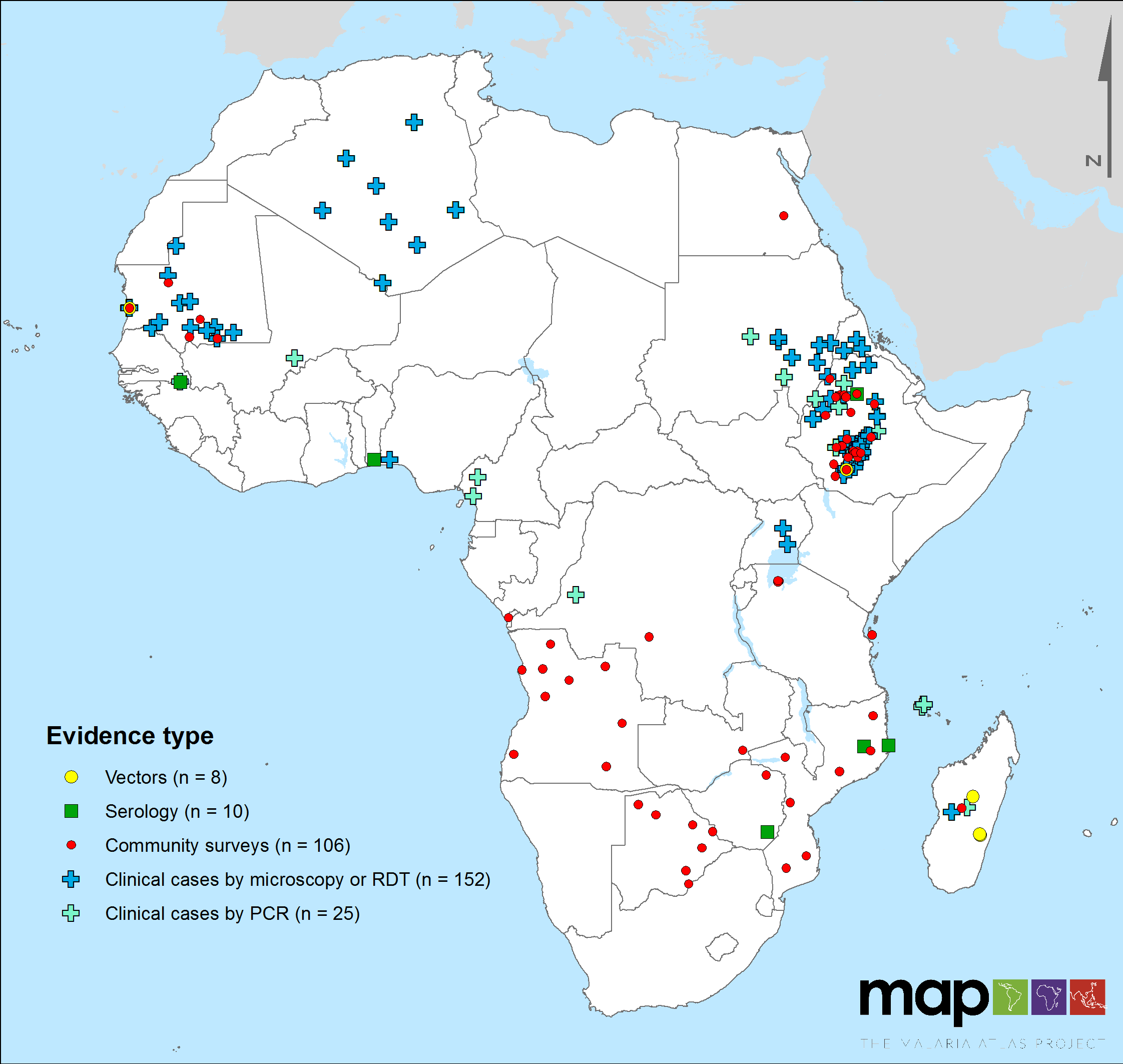

Supplement: S4 Fig — The spatial distribution and evidence class of all new reports of P. vivax occurrence added to the database since 2015 [14]. Data points are categorised into various evidence types: vectors (n = 8; yellow circles); serology (n = 10, green squares); community surveys from the recent literature review and MAP database (n = 106, red dots); and clinical cases (n = 177, blue crosses). (TIF) [file pntd.0007140.s006.tif]

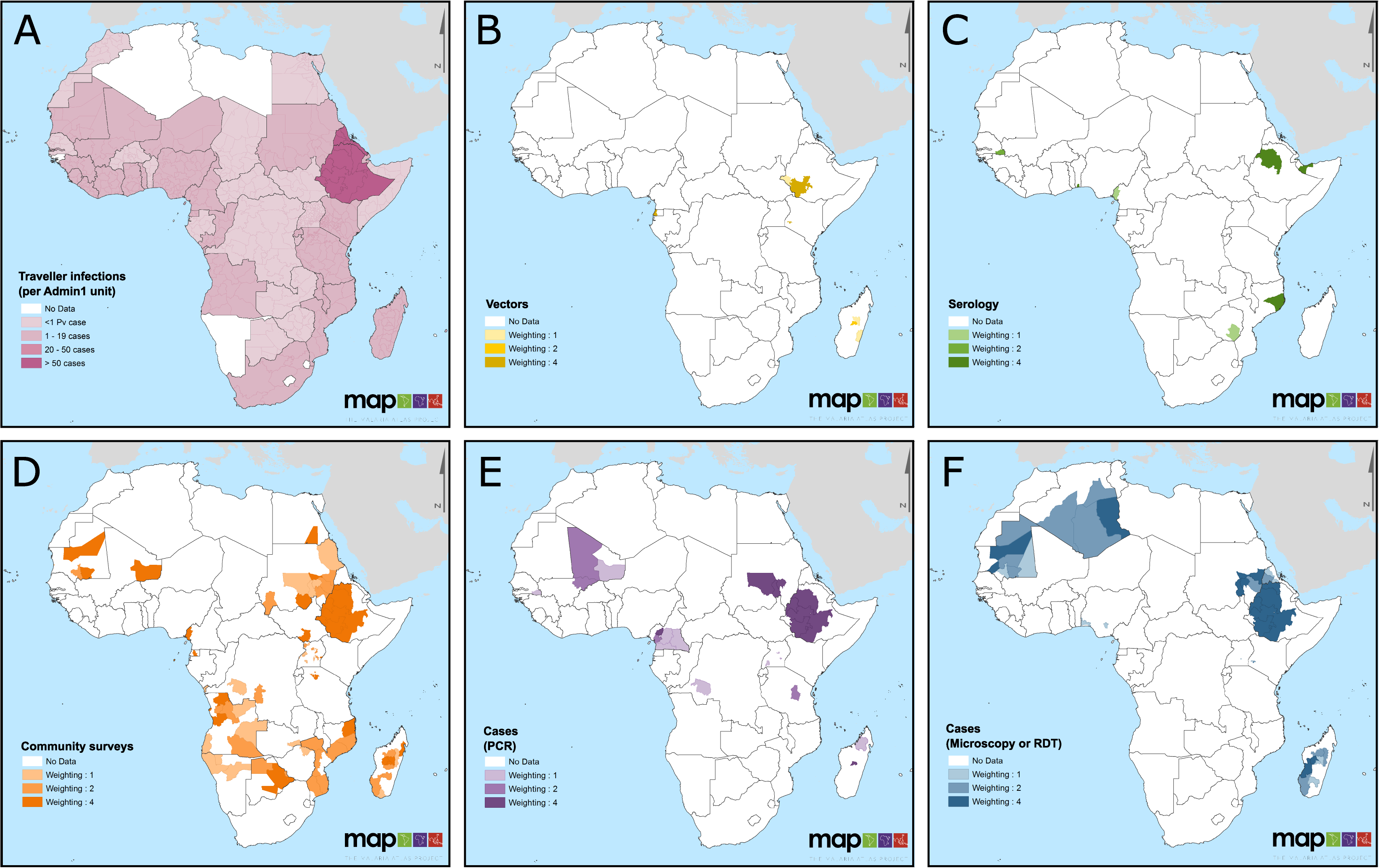

Supplement: S5 Fig — All evidence of P. vivax in Africa is summarised by strength and evidence class and displayed at the first-order administrative level (Admin1, e.g. state, province). Geopositioned reports of P. vivax were weighted according to Howes’ weighting framework (with modifications of the traveller weights) and assigned to the nearest Admin1 unit. Different panels depict distinct evidence classes (Panel A (pink)—admin-scaled number of infections among returning travellers; Panel B (yellow)—evidence of infected vectors; Panel C (green)—serological evidence of P. vivax infection; Panel D (orange)—community surveys detecting P. vivax infections; Panel E (purple)—local clinical cases diagnosed by PCR; Panel F (blue)—local clinical cases diagnosed by microscopy or RDT. Colour intensity indicates strength of weighted evidence. Traveller infections had an additional weighting category as follows: >40 traveller infections per Admin1 unit—weighting of 4; 25–40 traveller infections—weighting of 2, 1–25 traveller infections—weighting of 1; <1 traveller infection—weighting of 0.5. Refer to Fig 1C for full weighting criteria. (TIF) [file pntd.0007140.s007.tif]

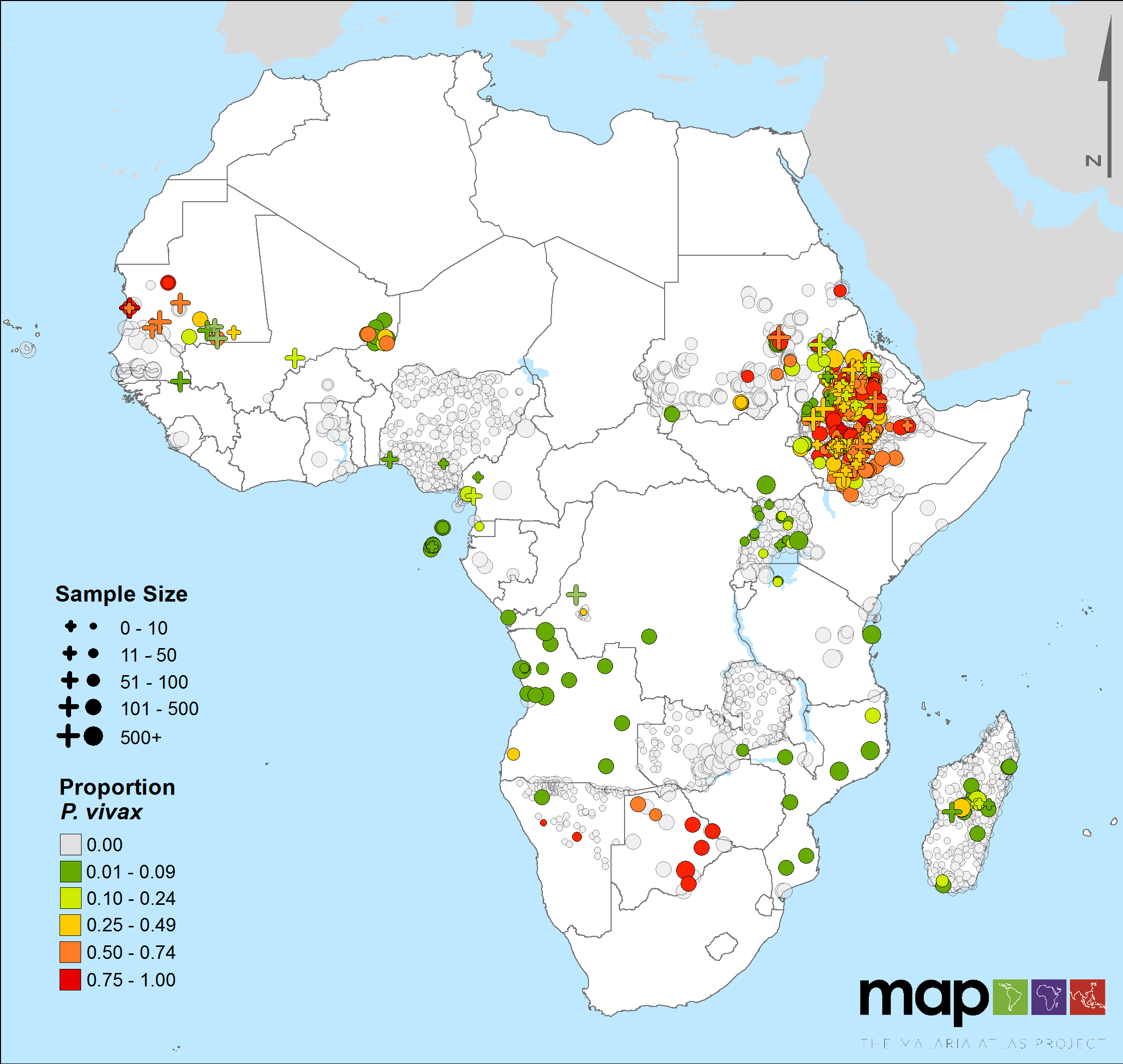

Supplement: S6 Fig — As in Fig 3A, a subset of sources from the final database that tested for both P. vivax and P. falciparum, and employed representative sampling methods are depicted. Prevalence points (round dots) and reports of local clinical cases (crosses) are coloured according to the proportion of infections due to P. vivax. Here, points are sized according to the total number of individuals examined at each site. (TIF) [file pntd.0007140.s008.tif]

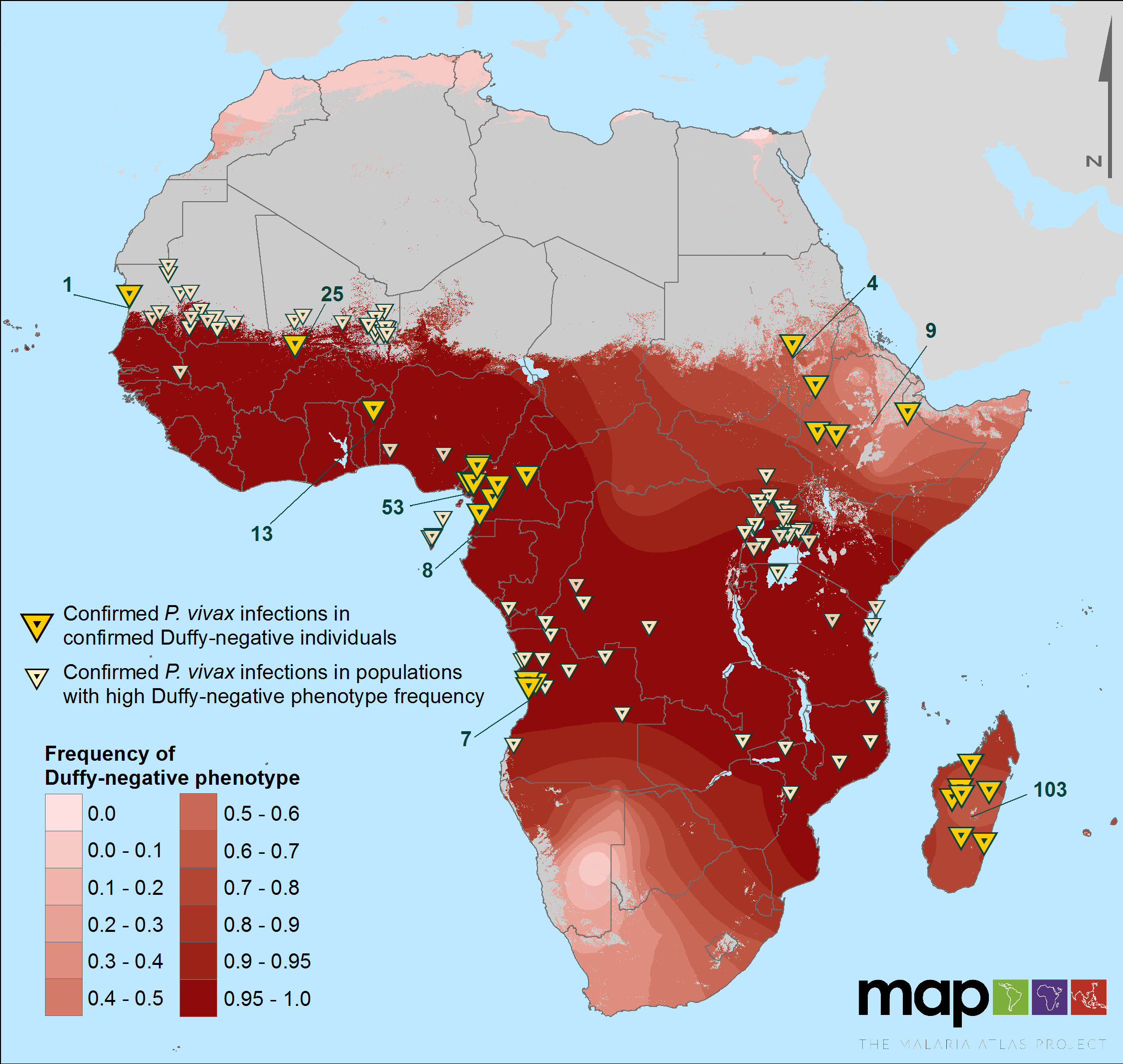

Supplement: S7 Fig — Dark yellow triangles indicate P. vivax infections in individuals with a confirmed Duffy-negative phenotype, with labels indicating national totals. Reports of any P. vivax infection in an area where the Duffy-negative phenotype is near fixation (frequency ≥0.95) are shown as light yellow triangles. The underlying map represents a modelled frequency map of the Duffy-negative phenotype across Africa (see [34]). Grey shading identifies areas within Africa where environmental conditions are unsuitable for P. vivax transmission [22]. (TIF) [file pntd.0007140.s009.tif]
