# Supplementary material for: Correction: Growing evidence of Plasmodium vivax across malaria-endemic Africa
Source: PLoS Negl Trop Dis. 2019 Jun 28;13(6):e0007525. doi: 10.1371/journal.pntd.0007525 (PMC6599138; doi:10.1371/journal.pntd.0007525)
Supplement: S2 File — (PDF) [file pntd.0007525.s002.pdf]

PLOS | NEGLECTED TROPICAL DISEASES

RESEARCH ARTICLE

Growing evidence of *Plasmodium vivax* across malaria-endemic Africa

Katherine A. Twohig[1]*, Daniel A. Pfeffer[1,2], J. Kevin Baird[3,4], Ric N. Price[2,4], Peter A. Zimmerman[5], Simon I. Hay[6], Peter W. Gething[1], Katherine E. Battle[1], Rosalind E. Howes[1]*

1 Malaria Atlas Project, Big Data Institute, Nuffield Department of Medicine, University of Oxford, Oxford, United Kingdom, **2** Global and Tropical Health Division, Menzies School of Health Research and Charles Darwin University, Darwin, Australia, **3** Eijkman-Oxford Clinical Research Unit, Eijkman Institute of Molecular Biology, Jakarta, Indonesia, **4** Centre for Tropical Medicine and Global Health, Nuffield Department of Medicine, University of Oxford, Oxford, United Kingdom, **5** The Center for Global Health & Diseases, Case Western Reserve University, Cleveland, Ohio, United States of America, **6** Institute for Health Metrics and Evaluation, University of Washington, Seattle, Washington, United States of America

* kate.twohig@bdi.ox.ac.uk (KAT); rosalind.howes@bdi.ox.ac.uk (REH)

Check for updates

OPEN ACCESS

Citation: Twohig KA, Pfeffer DA, Baird JK, Price RN, Zimmerman PA, Hay SI, et al. (2019) Growing evidence of *Plasmodium vivax* across malaria-endemic Africa. PLoS Negl Trop Dis 13(1): e0007140. https://doi.org/10.1371/journal.pntd.0007140

Editor: Marcus VG Lacerda, Fundacao Oswaldo Cruz, BRAZIL

Received: October 10, 2018

Accepted: January 7, 2019

Published: January 31, 2019

Copyright: © 2019 Twohig et al. This is an open access article distributed under the terms of the Creative Commons Attribution License, which permits unrestricted use, distribution, and reproduction in any medium, provided the original author and source are credited.

Data Availability Statement: Most relevant data are within the manuscript and its Supporting Information files. Data on imported malaria cannot be shared publicly because of various national data sharing laws, but should be available to researchers who meet the criteria for access to confidential data. Australian data are available from the Australian Government Department of Health National Notifiable Disease Surveillance System (contact via epi@health.gov.au). European country data are available from the ECDC European Surveillance System-TESSy (contact via Data.

Abstract

Effective malaria control strategies require an accurate understanding of the epidemiology of locally transmitted *Plasmodium* species. Compared to *Plasmodium falciparum* infection, *Plasmodium vivax* has a lower asexual parasitaemia, forms dormant liver-stages (hypnozoites), and is more transmissible. Hence, treatment and diagnostic policies aimed exclusively at *P*. *falciparum* are far less efficient against endemic *P*. *vivax*. Within sub-Saharan Africa, malaria control programmes justly focus on reducing the morbidity and mortality associated with *P*. *falciparum*. However, the recent emphasis on malaria elimination and increased accessibility of more sensitive diagnostic tools have revealed greater intricacies in malaria epidemiology across the continent. Since 2010, the number of studies identifying *P*. *vivax* endemic to Africa has expanded considerably, with 88 new scientific reports published since a review of evidence in 2015, approximately doubling the available data. There is evidence of *P*. *vivax* in all regions of Africa, apparent from infected vectors, clinical cases, serological indicators, parasite prevalence, exported infections, and *P*. *vivax*-infected Duffy-negative individuals. Where the prevalence of microscopic parasitaemia is low, a greater proportion of *P*. *vivax* infections were observed relative to *P*. *falciparum*. This evidence highlights an underlying widespread presence of *P*. *vivax* across all malaria-endemic regions of Africa, further complicating the current practical understanding of malaria epidemiology in this region. Thus, ultimate elimination of malaria in Africa will require national malaria control programmes to adopt policy and practice aimed at all human species of malaria.

Author summary

Plasmodium vivax malaria presents challenges for malaria elimination distinct from those of the other human malarias. Patients become symptomatic at lower asexual parasitaemia, making it harder to diagnose, and its dormant stages in the liver give rise to multiple

access@ecdc.europa.eu). United States data are available from the CDC National Malaria Surveillance System (contact via igd3@cdc.gov).

Funding: This works was funded by the Bill and Melinda Gates Foundation's grant to the ROAD-MAP (Repository of Open Access Data – Malaria Atlas Project) group within MAP (grant OPP1106023). Simon I. Hay was primarily supported by grant OPP1132415 by the Bill & Melinda Gates Foundation. https://www.gatesfoundation.org/ The funders had no role in study design, data collection and analysis, decision to publish, or preparation of the manuscript.

Competing interests: The authors have declared that no competing interests exist.

clinical attacks unless treated with an 8-aminoquinoline drug. The conspicuous dominance of *P. falciparum* across most of malaria-endemic Africa has historically obscured other malarias in national policies. The literature reviewed here expands a prior systematic examination of the evidence of *P. vivax* transmission in Africa. Analysis of the relative proportions of infections due to *P. vivax* in relation to overall malaria prevalence suggests an increased *P. vivax* ratio in areas of relatively low transmission. In addition, while studies demonstrate a protective effect of Duffy-negativity against the risk of *P. vivax* infection, this highly prevalent African blood group does not always prevent that infection altogether. As the burden of *P. falciparum* declines with conventional control success, transmission of the other malarias may become more obvious and important as major hurdles for elimination. Acknowledging and identifying the reservoirs enabling transmission of *P. vivax* begins to enable effective countermeasures to be devised and deployed.

Introduction

Significant funding increases for global malaria control between 2005 and 2010 facilitated a rapid decrease in the burden of malaria, with an estimated 37% reduction in case incidence and a 60% drop in mortality from 2000 to 2015 [1]. During this period, malaria control programmes focused on scaling-up coverage of key interventions such as insecticide-treated bed nets, effective antimalarial drugs, and point-of-care diagnostics, together with an expansion of fundamental and operational research [1, 2]. History demonstrates the logical and salient relationship between financial resources and disease burden, with diminished funding resulting in rapid resurgence of case numbers [3, 4]. At the global level, recent financial pressures have stalled the availability of funds for malaria interventions, and slowed progress in reducing malaria burden since 2013 [5]. The reality of finite funding highlights the crucial importance of rationally targeted interventions appropriate to the underlying biology and epidemiology of the etiologic agent.

Among the most fundamental determinants of appropriately targeted malaria control interventions are the biology of the vector and parasite species, the possible combinations of which number many hundreds [6]. Vector behaviours and capacity to transmit malaria differ significantly among the malaria-transmitting *Anopheles* species, as does the vulnerability of *Plasmodium* parasites to specific control strategies. A key example of differential response to interventions is the treatment of *Plasmodium vivax* [7, 8]. This parasite's life cycle differs from that of *Plasmodium falciparum* by having dormant liver stage hypnozoites, which can later awaken to provoke repeated infection relapses in the weeks, months, and several years following an initial inoculation. This latency is unknown in *P. falciparum* malaria. Hypnozoites are unaffected by blood-stage therapies, including artemisinin-combination therapies (ACTs), the first-line treatment for acute malaria in most countries. Subsequently, morbidity and mortality associated with acute and chronic *P. vivax* infections continue where this parasite is present and no specific treatment against hypnozoites is applied [9, 10]. Thus the management of endemic transmission of vivax malaria requires inclusion of a haemolytically-toxic 8-aminoquinoline "radical cure" that kills hypnozoites [11].

Until relatively recently, *P. vivax* was rarely studied across most of sub-Saharan Africa. The overwhelming dominance of the Duffy-negative blood group has been regarded as a key determinant preventing the sustained endemic transmission of *P. vivax* through much of the continent [12]. This inherited trait means that red blood cells lack the Duffy receptor, an important molecule for *P. vivax* parasite invasion. However, recent studies have demonstrated that

P. *vivax* infection can still occur in Duffy-negative red blood cells [13–15], and thus Duffy-negativity alone may not prevent endemic transmission. A review of the evidence of *P*. *vivax* transmission across Africa published in 2015 [14] supported that assessment. Since then, a large number of new studies have reported additional evidence (S1 Fig). In this report, we collate the up-to-date data on *P*. *vivax* transmission in Africa, producing a qualitative evidence strength map and employing a consistent methodological approach to the previous iteration that allows for comparison. While *P*. *falciparum* malaria conspicuously dominates most sub-Saharan countries, assembling documented evidence of other malaria species serves the important need of assessing real and present obstacles to achieving malaria elimination. Recent escalating research interest furthers our understanding of the significance of *P*. *vivax* across this diverse continent, providing evidence with which to target malaria control strategies of greater scope and economy of effort.

Methods

Updating the evidence base of *Plasmodium vivax* transmission in Africa

To build on the database of evidence compiled in Howes *et al*. 2015 [14], a literature review was undertaken to bridge the period from 1/12/14 to 25/04/18 and identify additional reports of *P*. *vivax* throughout Africa (S2 Fig). The search was carried out in PubMed with keywords "vivax" AND "[African country name]", and "vivax" AND "Africa". The new literature was assessed against the same categories as previously [14]: locally-diagnosed clinical cases, serological studies, infected *Anopheles* vectors, cross-sectional community prevalence surveys, and reports of imported *P*. *vivax* malaria into non-endemic countries. Data were extracted from each paper and geopositioned at the highest geographic resolution given. To supplement this search, reference lists were consulted for additional data sources and the Malaria Atlas Project parasite rate (PR) and annual parasite incidence (API) databases were queried for records of *P*. *vivax* [16–18]. Finally, surveillance reports of imported malaria into non-endemic countries were reviewed for aggregated annual case numbers by probable African country of infection and malaria species. These were assembled from openly accessible sources and by direct contact with disease surveillance programmes (S2 Fig).

For consistency, Howes *et al*.'s previous evidence weighting framework was applied to the new data, with a small modification to the returning traveller weights to make the lowest category more conservative (from 20 to 25 infections) and the highest slightly less conservative (from 50 to 40 infections). R version 3.3.3 and R packages tidyverse [19], rdgal [20] and sp [21] were used to apply the weighting framework to the consolidated dataset and determine the strength of evidence for each data type at the sub-national administrative level 1 ("Admin1", i.e. state/province) throughout Africa. As described in Howes *et al*., the weighted evidence categories aimed to reflect the relative strengths of different diagnostic methods and study designs for each data type and to provide a qualitative relative assessment of the evidence of *P*. *vivax* transmission [14]. Point-specific data provided evidence for each associated Admin1 unit, while total national traveller cases were divided by the number of Admin1 units in each country. Weighting of the traveller data did not distinguish between the diagnostic methods employed as those details were not available for all datasets. Thus, the traveller data was less specific and that category was down-weighted relative to the other data types. The sum of weighted evidence from each category was then mapped at the Admin1 level using ESRI ArcMap 10.6. The spatial limits of environmental suitability for *P*. *vivax* transmission across Africa [22] were applied to the final evidence summaries to reflect climatic heterogeneity within Admin1 units. The shapefiles used for all maps were curated by the Malaria Atlas Project [18]

using GADM datasets [23], with updates performed where sources suggested boundaries have changed.

Assessing the relative importance of *Plasmodium vivax*

The clinical case reports and prevalence surveys were evaluated to determine whether they offered a representative estimate of site-level malaria parasite species proportions. Very few studies reported cases due to *Plasmodium ovale* or *Plasmodium malariae* so these species were excluded from species proportion estimates. The requirement for inclusion was that the study designs and reported results were unbiased to a particular parasite species, and could be assumed to be representative of the relative burdens of *P. vivax* and *P. falciparum* at that location. The assessment was based primarily on the sampling approach and diagnostic methods employed. This subset of clinical case and prevalence points was visualized to determine the geographic patterns of the proportion of *P. vivax* relative to the total malaria cases. A secondary component of this analysis explored the relationship between the *P. vivax* proportions and the overall *P. vivax* and *P. falciparum* prevalence from each survey.

Updating the database of *Plasmodium vivax* infections in Duffy-negative individuals

The current literature review also built upon our existing database of reported *P. vivax* infections in Duffy-negative individuals [14]. A systematic search in PubMed with the terms "vivax AND (Duffy OR CD234 OR DARC)" was conducted on 25/04/2018 to identify relevant reports published since 01/12/2014. Search results were cross-referenced against recent review papers [13, 15]. In contrast to these reviews, only papers that conclusively tested individuals for both *P. vivax* infection and Duffy-negative phenotype were included in the current database. Surveys for which both Duffy phenotype and *P. vivax* infection status were available for all individuals and at least one *P. vivax* infection was detected in both Duffy groups were analysed to calculate odds ratios of *P. vivax* infection in Duffy-negative individuals relative to Duffy-positives using Wald unconditional maximum likelihood estimation [24].

Results

Strength of evidence of *Plasmodium vivax* transmission in Africa

The current update to the evidence base covered publications from the last three years (December 2014 to April 2018). This identified new evidence from 177 local *P. vivax* clinical case reports at 96 sites (S1 Table) and 79 community surveys at 56 sites reporting the presence of *P. vivax* infections (S2 Table). There were eight new reports of *P. vivax*-infected *Anopheles* vectors (S3 Table) and ten of *P. vivax*-seropositivity (S4 Table). These updates, when combined with the previous evidence base, document *P. vivax* at 657 unique sites across 29 African countries (Fig 1A and S4 Fig), including from six previously undocumented endemic countries: Benin, Comoros, Mozambique, Senegal, Zambia and Zimbabwe. Updated versions of the summary figures included in the first publication [14] are available in the Supplementary Information (S5 Fig).

A total of 48,321 malaria infections believed to have been acquired in Africa but exported elsewhere by visiting travellers were also collated, representing an opportunistic dataset from accessible country reports and programme contacts, rather than any effort at comprehensiveness (Figs 1B and S3). These were diagnosed in 37 non-endemic countries as well as China, which has a surveillance programme that allows imported cases to be differentiated from locally-acquired infections [25]. Nearly 13% of the returning traveller cases from Africa

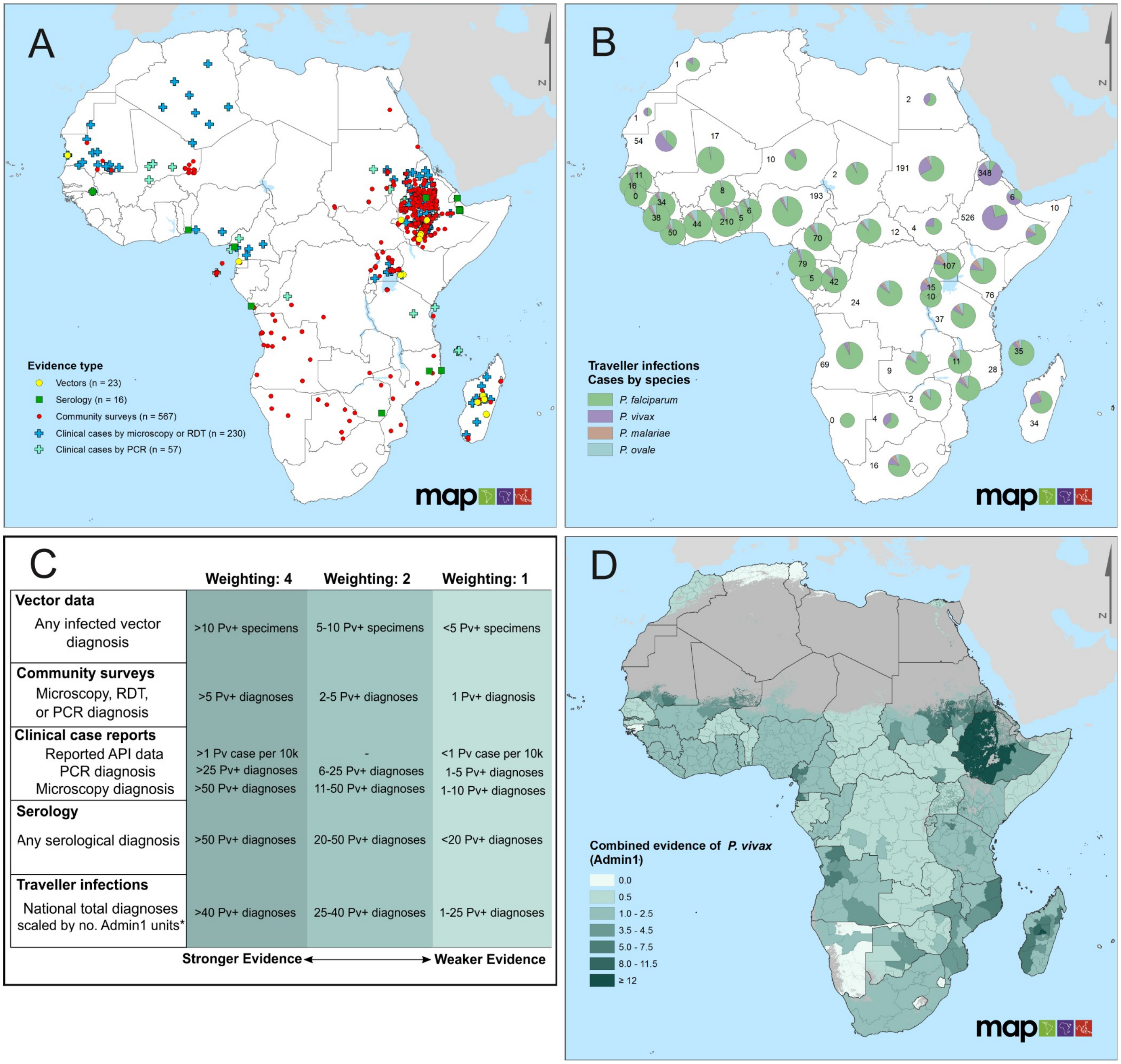

Fig 1. Evidence of *P. vivax* in Africa. Panel A maps all available reports of *P. vivax* occurrence (n = 657 unique sites) by evidence type (1980–2016). Data from Howes *et al.* [14] are combined with the results of this update (new points are shown in S4 Fig). Panel B summarises exported malaria infections among travellers returning to non-endemic countries, grouped by suspected country of origin and *Plasmodium* species. Pie charts represent the proportional contribution by malaria species, sized based on a log-transformation of the total number of infections. The numbers shown beside each pie chart refer to the number of *P. vivax* infection reports attributed to each country. The traveller infections database represents an opportunistic assembly of reports, and does not aim to be comprehensive; reports are aggregates of data from 1991 to 2016, see S3 Fig for timespan of input data. Panel C documents the framework used to weight the data from Panels A and B to characterise the strength of evidence of local *P. vivax* transmission. Evidence weights (top row) were assigned for each evidence type in each Admin1 unit (state/province), and summed to indicate the total evidence of *P. vivax* in a given unit. The resulting composite evidence map is shown in Panel D. Darker shading indicates increased evidence of the presence of *P. vivax.* White indicates units for which no evidence was found (evidence strength = 0), and grey shading indicates regions where environmental conditions are unsuitable for *P. vivax* transmission [22]. *National reported infections among returning travellers were scaled by the total number of Admin1 units in a given country. For countries with fewer traveller *P. vivax* cases than Admin1 units, a weighting of 0.5 was given to each admin unit.

https://doi.org/10.1371/journal.pntd.0007140.g001

reviewed for this study were due to non-falciparum species, with *P*. *vivax* infections alone representing 5% of the reviewed total (n = 2,473). Across the assembled *P*. *vivax* case reports, 44 African countries from throughout the continent were listed as suspected origins of infection, with seven countries attributed to more than 50 "exported" *P*. *vivax* cases since 2010. *Plasmodium vivax* represented the majority of traveller malaria infections in Ethiopia, Eritrea and Mauritania.

Given the diversity of evidence types assembled, each being associated with relative strengths and weaknesses as evidence of local transmission, these were differentially weighted according to the criteria given in Fig 1C, with the resulting subnational regional evidence strength plotted in Fig 1D and S5 Fig. The presence of strong evidence (≥ 5) was observed in 13 countries across the continent, with at least one Admin1 unit in all African Union regions except West. The strongest evidence of *P*. *vivax* was observed in the Eastern region, notably in Ethiopia, Madagascar, and Sudan, as well as Mauritania. No evidence was reported from 109 units, including the entirety of Cape Verde, Guinea-Bissau, Mayotte, and Swaziland, while the majority of areas with evidence were associated with the weakest evidence category, derived only from traveller infections (587/721, 81.4%). Nearly 15% of all Admin1 units (n = 119/830 from 26 countries) had evidence in at least two data categories.

Species ratios

Fig 2A presents a map of community prevalence surveys and clinical case points found to be representative indicators of the local species composition (n = 565 prevalence points with a

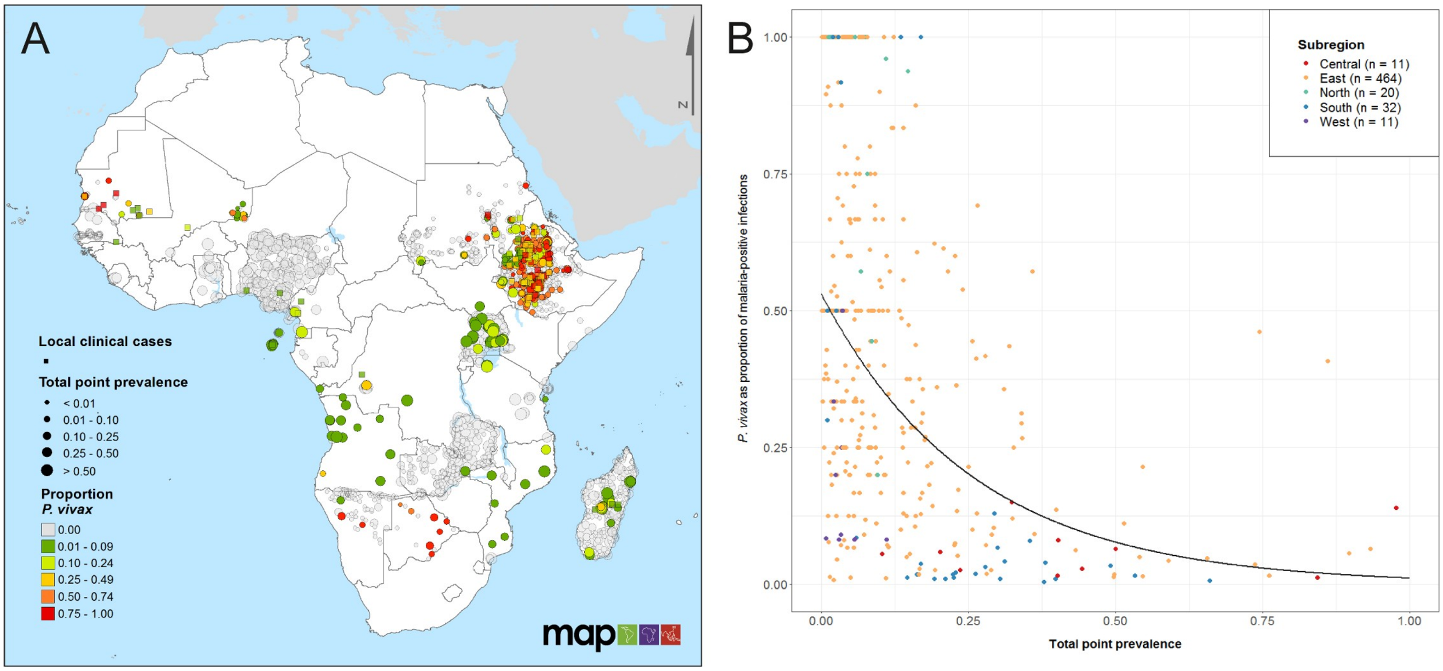

Fig 2. Relationship between proportion of infections due to *P. vivax* and total malaria prevalence across Africa. Panel A shows the spatial distribution of the proportion of *P*. *vivax* infections relative to *P*. *falciparum*, based on a subset of sources from the final database that tested for both *P*. *vivax* and *P*. *falciparum*, and employed representative sampling methods. Prevalence points (round dots, total n = 3,329; n = 565 with *P*. *vivax* proportion > 0) are coloured according to the proportion of infections due to *P*. *vivax*, and sized according to the total point prevalence at each site. Clinical case reports where the species proportion could be determined are represented as squares (n = 152) and also coloured by *P*. *vivax* proportion. S6 Fig shows the equivalent map with points sized by sample size. Panel B plots site-wise proportion of infections due to *P*. *vivax* (y-axis) in relation to overall community prevalence (x-axis) at locations where the total number examined was at least 30 individuals and at least one *P*. *vivax* infection was detected (n = 538). Only surveys that used diagnostic methods sensitive to both *P*. *vivax* and *P*. *falciparum* were included. Points are coloured according to African Union regions (S1 Fig) [47], with trend lines added as a visual guide.

https://doi.org/10.1371/journal.pntd.0007140.g002

P. *vivax* proportion greater than zero; n = 152 clinical case points). These locations signified studies where diagnostic tests sensitive to *P*. *vivax* and *P*. *falciparum* were used and species-specific infections were reported. Studies where no *P*. *vivax* parasites were found (n = 3,329) were also mapped, indicating widespread reporting of apparent zero prevalence of *P*. *vivax* [26]. Many of the countries with high spatial coverage of points, including Zambia, Ethiopia, Nigeria and Uganda, indicate the sampling efforts of recent Malaria Indicator Surveys where species-specific diagnostics were used [27, 28]. Where there were both clinical case and prevalence points at a similar location, the *P*. *vivax*-proportion was mostly consistent between the two data types (Fig 2A). It is important to note that there is considerable variation in the number of examined individuals for each of these data points, for example, 75.4% of points shown in Uganda and 87.5% in Nigeria had a sample size of less than 30 individuals (S6 Fig).

Fig 2B explores the association between total parasite rate and the proportion of infections due to *P*. *vivax* using the subset of points from Fig 2A, where at least 30 individuals had been tested for malaria and at least one *P*. *vivax* infection was reported (n = 538). Two thirds of the points (70.8%; n = 381) had a total prevalence of less than 0.1, and more than two thirds of those had a *P*. *vivax* proportion greater than 50%. Among data points where total parasite rate was greater than 0.5, very few had *P*. *vivax* proportions exceeding 25%. There were 74 points outside of the East region; forty percent of these had *P*. *vivax* proportions greater or equal to 25%. Regions with lower overall malaria prevalence had the highest mean *P*. *vivax* proportions.

Plasmodium vivax infection in relation to Duffy phenotype

Nine new reports of *P*. *vivax* positivity of Duffy-negative individuals were identified since the first version of this evidence assembly, contributing to an overall total of 223 infected individuals in nine African countries (S5 Table). The assembled evidence highlights the occurrence of *P*. *vivax*-infected Duffy-negative hosts across areas where Duffy-negativity is at near fixation, both as asymptomatic and clinical *P*. *vivax* infections (Fig 3). *Plasmodium vivax* infections have also been identified in populations estimated to have very high ($\geq$95%) Duffy-negativity from studies where individual-level Duffy phenotype was not confirmed ($N_{locations}$ = 266; S7 Fig). Fig 3B illustrates the odds of *P*. *vivax* infection in Duffy-negative relative to Duffy-positive individuals. In each of the five studies for which an odds ratio (OR) could be calculated (Fig 3B), the odds of Duffy-negative individuals being positive for *P*. *vivax* were significantly lower than for Duffy-positive individuals (OR (95% CI) $<$1). The odds of infection in Duffy-negatives were significantly lower in clinic-based studies (presumably mainly symptomatic patients) than in cross-sectional community surveys (likely mainly asymptomatic infections). Infection status was determined by either PCR or microscopy (S5 Table).

Discussion

As goals for malaria elimination in southern Africa draw closer to success and other countries across the continent strive to approach pre-elimination status, research on the non-falciparum malarias has seen marked growth, notably outside of the traditionally held 'Eastern' region (S1 Fig). In this update, more than a third (38%, 37/96) of sources provided data from the rest of the continent, compared with less than 20% in the initial review. Furthermore, recent studies (since 2014) provided more than 60% of that data. It is evident that greater importance is now being placed upon non-falciparum malaria species within contemporary malaria research in Africa than ever before. Nevertheless, according to the 2017 World Malaria Report [5], 38 of 47 African countries reported that 100% of their malaria infections were attributable only to *P*.

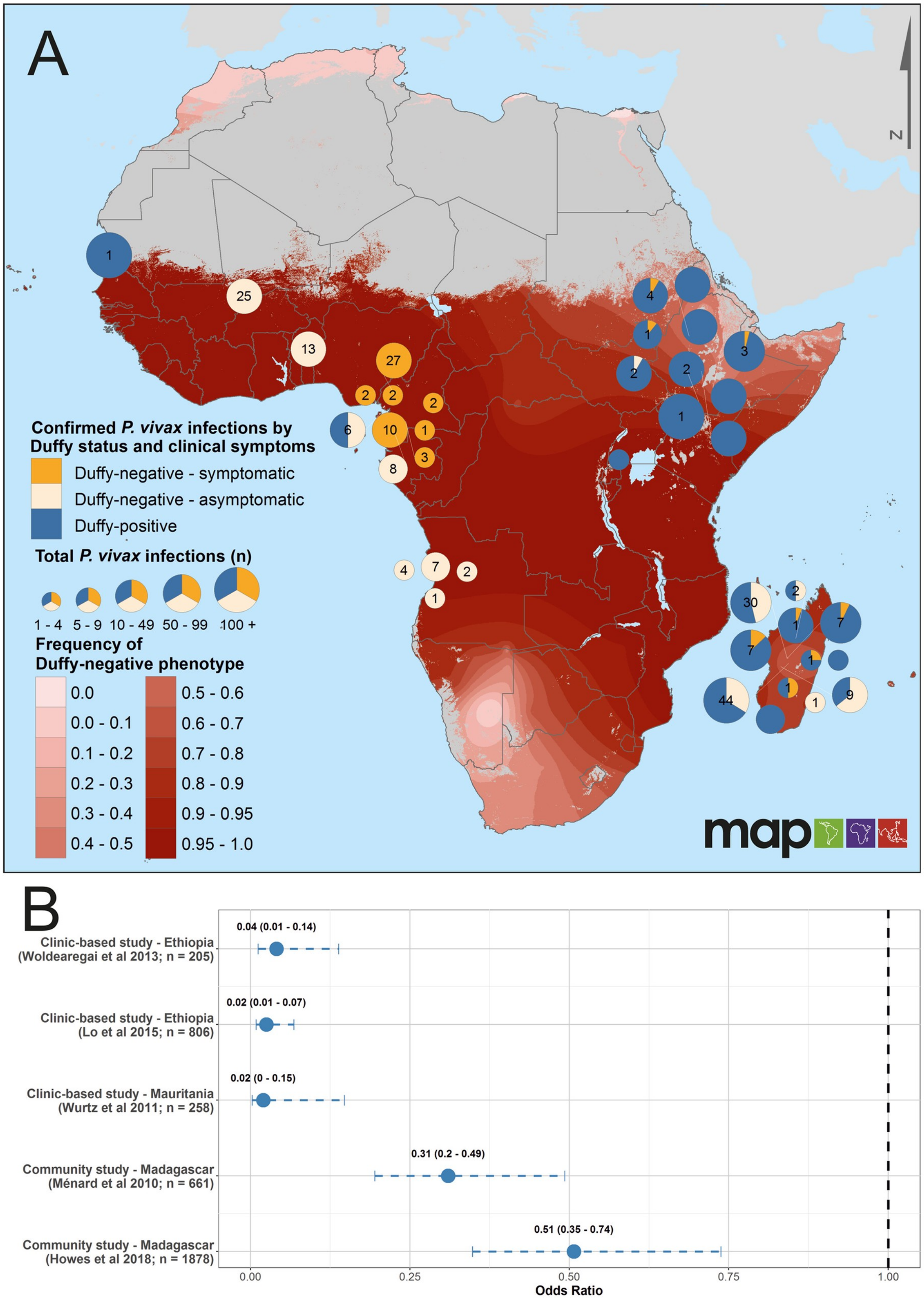
A
N
Confirmed P. vivax infections by
Duffy status and clinical symptoms
Duffy-negative - symptomatic
Duffy-negative - asymptomatic
Duffy-positive
Total P. vivax infections (n)
1 - 4
5 - 9
10 - 49
50 - 99
100 +
Frequency of
Duffy-negative phenotype
0.0
0.0 - 0.1
0.1 - 0.2
0.2 - 0.3
0.3 - 0.4
0.4 - 0.5
0.5 - 0.6
0.6 - 0.7
0.7 - 0.8
0.8 - 0.9
0.9 - 0.95
0.95 - 1.0
map
B
Clinic-based study - Ethiopia
(Woldearegai et al 2013; n = 205)
0.04 (0.01 - 0.14)
Clinic-based study - Ethiopia
(Lo et al 2015; n = 806)
0.02 (0.01 - 0.07)
Clinic-based study - Mauritania
(Wurtz et al 2011; n = 258)
0.02 (0 - 0.15)
Community study - Madagascar
(Ménard et al 2010; n = 661)
0.31 (0.2 - 0.49)
Community study - Madagascar
(Howes et al 2018; n = 1878)
0.51 (0.35 - 0.74)
0.00
0.25
0.50
0.75
1.00
Odds Ratio

Fig 3. *Plasmodium vivax* positivity in Duffy-negative individuals in Africa. Panel A summarises proportions of detected *P. vivax* infections among individuals who are Duffy-negative (orange) relative to those who are Duffy-positive (blue), by site. Different shades of orange distinguish symptomatic (clinic-based studies; dark orange) and asymptomatic (community studies; light orange) *P. vivax* infections in Duffy-negative individuals. Pie charts are sized relative to the total individuals tested for both *P. vivax* and Duffy status, with numbers corresponding to the total numbers of *P. vivax* infections of Duffy negative hosts. Grey shading illustrates areas in Africa with environmental conditions unsuitable for *P. vivax* transmission [22]. Panel B plots the odds ratios (OR) of *P. vivax* infection among Duffy-negative vs Duffy-positive individuals, in a subset of surveys that reported the Duffy phenotype and *P. vivax* infection status for all individuals, and where *P. vivax* infections were observed in both Duffy phenotypes. Infection counts were summed from all locations in each study.

https://doi.org/10.1371/journal.pntd.0007140.g003

falciparum. Diagnostics frequently remain limited to *P. falciparum*, with only 20 of 47 African countries reporting the use of rapid diagnostics sensitive to the other *Plasmodium* species in 2017 [5]. There may often be a disconnect between the parasites being locally transmitted and the diagnostics applied to detect them, leading to misreporting and misrepresentation of endemic malaria species occurring locally [29]. In this study, we emphasize the importance of *P. vivax*, confident in the knowledge that strategies that target both *P. falciparum* and *P. vivax* are likely to provide full efficacy against all species (i.e. including *Plasmodium malariae* and *Plasmodium ovale*) in a way that current policies targeting solely or principally acute *P. falciparum* malaria do not [6, 30].

As previously discussed [14], it is important to note that observations reported in this data assembly may be susceptible to misdiagnosis or be erroneously considered evidence of local transmission. Diagnostic confounders such as morphological similarity to *P. ovale* [31], proximity to zoonotic reservoirs of *P. vivax*-like parasites [32] and delayed onset of relapsing infections not locally acquired [33] might influence the observed evidence of vivax malaria presence in Africa. To account for these possible alternative explanations, the categories of evidence defined here are deliberately weighted conservatively (Fig 1C) and favour more sensitive diagnostics. Furthermore, misdiagnosis of *P. ovale* infections or relapses from infections inoculated elsewhere nevertheless constitute a public health burden requiring appropriate response at the geographic locations of diagnosis.

Plasmodium vivax in Africa is widespread, diverse, and likely often undetected

Consolidating publicly available records of confirmed *P. vivax* parasitaemia demonstrates the compelling evidence of widespread transmission of *P. vivax* across Africa. Reports from 29 countries describe local *P. vivax* clinical cases, infected vectors or asymptomatic parasitaemia, demonstrating an endemic range extending well beyond the Eastern region and penetrating areas of very high (>95%) Duffy-negativity, from where *P. vivax* was previously thought to be absent [22, 34, 35]. The documented presentation of *P. vivax* infections across Africa is diverse and context-specific, driven by the specific objectives of isolated clinical or epidemiological activities. The varied diagnostics and methodological approaches used across studies limit our ability to concretely infer distinct epidemiological characteristics of *P. vivax* between regions. More systematic use of sensitive point-of-care diagnostics or molecular tools in community prevalence surveys might enable such insights in the future.

Plasmodium vivax in Africa is reported at higher proportions in locations with lower malaria burden

Our analysis reveals that *P. vivax* is proportionally more significant where overall malaria prevalence is lower (Fig 2). There have been reports in co-endemic settings of shifts in relative parasite composition as overall malaria burden decreases [30, 36]. However, countries are

inherently more likely to enhance their diagnostic capacity (including testing for non-falciparum species) when entering elimination phases: it is unclear whether the relationship observed in Fig 2 was due to changing transmission patterns or was confounded by heightened awareness and surveillance efforts in pre-elimination settings. The trends in the available data nevertheless appear to support the status of *P*. *vivax* as a more resilient challenge to elimination [6].

The Duffy-negative phenotype is not a definitive barrier to *P*. *vivax* infection

Assumptions regarding the general absence of *P*. *vivax* in Africa [22, 34] were based on the high prevalence of Duffy-negativity across much of the continent [12, 35]. In recent years however, a growing body of evidence has documented *P*. *vivax* infections in confirmed Duffy-negative individuals across much of Africa, totalling 223 symptomatic and asymptomatic infections across nine countries (Fig 3 and S5 Table). Furthermore, reports of *P*. *vivax* infections in populations with a high estimated Duffy-negative phenotype frequency (>95%) suggests the likely occurrence of additional cases (S6 Fig) [15] and ongoing transmission sustained even in populations with very few Duffy-positive hosts [14].

While not a definitive barrier, it is nonetheless evident that Duffy-negativity offers significant protection against *P*. *vivax* blood-stage infection, notably in symptomatic patients presenting for treatment (Fig 3B). This aligns with long-prevailing thinking regarding the Duffy antigen as an important component of *P*. *vivax* invasion [37]. Several additional host cell receptors (e.g., CD71 and CD98) have recently been identified as being involved in the parasite invasion pathway of red blood cells [38, 39]. Absence of the Duffy antigen appears to render the invasion mechanism less efficient, but not null. Understanding host-parasite invasion junctions may allow more specific assessments of the risks of *P*. *vivax* infection and clinical disease across the Duffy-negative populations previously considered fully protected, as well as identifying potential vaccine targets.

Plasmodium vivax imposes complexity to malaria elimination in Africa

The evidence presented here illustrates the widespread distribution of *P*. *vivax* across the whole of malaria-endemic Africa. It demonstrates that the overall contribution of *P*. *vivax* to morbidity associated with malaria is much smaller than that attributable to *P*. *falciparum*, but that that contribution increases in areas of lower endemicity. The diverse epidemiological settings in which *P*. *vivax* malaria has been detected indicates that awareness of the geographic distribution and epidemiology of this parasite across Africa may be vital to shape strategies aiming to eliminate all malarias from the continent, and that elimination efforts may have to incorporate *P*. *vivax*-specific interventions in order to achieve this goal. This challenge is evident in the case of Botswana. The past decade has seen a dramatic decline in malaria cases nationwide, yet elimination has remained elusive [40]. One key aspect to this problem remains comprehensive diagnosis, with most cases identified via passive surveillance using RDTs unable to diagnose non-*P*. *falciparum* parasites [41, 42]. As Motshoge *et al*. report, recent national surveys reveal a significant asymptomatic reservoir of *P*. *vivax* that remains undetected by routine surveillance [43], sparking an interest in introducing primaquine to target this *P*. *vivax* reservoir [44]. Low-density and asymptomatic *P*. *vivax* infections have been shown capable of maintaining transmission through the presence of mature gametocytes [45, 46]. Unless *P*. *vivax*-sensitive diagnostics and latent liver-stage therapies are employed, it seems unlikely that this silent and invisible reservoir of *P*. *vivax* will be effectively cleared.

Significant reductions in the global malaria burden, particularly in Africa, have thus far been achieved using generic commodities like RDTs, ACTs, and insecticide-treated bed nets.

However, those gains have stabilized and plateaued [5]. The asymptomatic, sub-patent, and latent malarias of all of the *Plasmodium* species that infect humans will harshly test our tools, resources, and dedication on the path to an envisioned elimination. Priorities for malaria control resources are justly allocated to the species dominating associated morbidity and mortality, but realisation of temporally and geographically dynamic shifts in that dominance can rationally inform such allocations and their optimal form. Documenting species-specific evidence facilitates this assessment. To achieve malaria elimination, strategists and workers in Africa will eventually have to deal with all of these malarias, including an apparently widespread and diverse underlying *P. vivax* malaria problem.

Supporting information

S1 Checklist. PRISMA checklist.
(DOC)

S2 Checklist. PRISMA flow diagram.
(DOC)

S1 Fig. Timeline of extracted data sources reporting *P. vivax* presence throughout Africa. The number of sources (y-axis) by year of publication (x-axis). Bars are coloured according to the African Union region [47] from which data in each source originated (shown in inset map). The dashed line approximately indicates the data sources included in Howes *et al.* 2015; sources after the line have been added since that publication. Excluded from this plot are nine additional articles from the beginning of 2018 (through to the search date 25/04/2018), and one unpublished report. Occasionally sources would report data from multiple countries and multiple regions, in which case an individual source was counted more than once. However, duplicates were removed to the extent possible to show distinct sources. All unique combinations of source, publication year and region were retained.
(TIF)

S2 Fig. Schematic overview of the collation of evidence of *P. vivax* transmission in Africa. Data was sourced from a comprehensive literature search, the Malaria Atlas Project's malariometric databases, and contributions of data on imported traveller infections from national/regional infectious disease surveillance programs outside of Africa. Evidence types were categorised, and new data (blue) was combined with data from Howes *et al.* (red) to yield a final updated evidence base (purple). The number of reports (n reports) refers to the number of spatially and temporally unique points. It is not the same as the number of unique geographic locations. In five instances, evidence was found for multiple data types in the same source. Thus, the number of new unique sources (blue) is 96. The 2018 literature search included all African countries, unlike the previous 2015 search which was limited to countries endemic with malaria in 2014.
* The traveller database was updated with additional years of data from the same source in one instance, so the total number of sources is not strictly the sum of all separate contributions.
(PNG)

S3 Fig. Summary of returning traveller infection database. Data on returning traveller infections was mainly collected from national surveillance programmes through data access requests, personal communications, publicly accessible reports and scientific publications of aggregate data. Other data were obtained through the literature search and mainly consisted of case reports not included in the national aggregates.
(TIF)

S4 Fig. Summary of new evidence published since 2015 of *P. vivax* occurrence, indicated by data type. The spatial distribution and evidence class of all new reports of *P. vivax* occurrence added to the database since 2015 [14]. Data points are categorised into various evidence types: vectors (n = 8; yellow circles); serology (n = 10, green squares); community surveys from the recent literature review and MAP database (n = 106, red dots); and clinical cases (n = 177, blue crosses).
(TIF)

S5 Fig. Admin1-level weighted evidence of *P. vivax* in Africa, according to evidence class. All evidence of *P. vivax* in Africa is summarised by strength and evidence class and displayed at the first-order administrative level (Admin1, e.g. state, province). Geopositioned reports of *P. vivax* were weighted according to Howes' weighting framework (with modifications of the traveller weights) and assigned to the nearest Admin1 unit. Different panels depict distinct evidence classes (Panel A (pink)—admin-scaled number of infections among returning travellers; Panel B (yellow)—evidence of infected vectors; Panel C (green)—serological evidence of *P. vivax* infection; Panel D (orange)—community surveys detecting *P. vivax* infections; Panel E (purple)—local clinical cases diagnosed by PCR; Panel F (blue)—local clinical cases diagnosed by microscopy or RDT. Colour intensity indicates strength of weighted evidence. Traveller infections had an additional weighting category as follows: >40 traveller infections per Admin1 unit—weighting of 4; 25–40 traveller infections—weighting of 2, 1–25 traveller infections—weighting of 1; <1 traveller infection—weighting of 0.5. Refer to Fig 1C for full weighting criteria.
(TIF)

S6 Fig. Spatial distribution of the proportion of *P. vivax* infections relative to *P. falciparum* in Africa. As in Fig 3A, a subset of sources from the final database that tested for both *P. vivax* and *P. falciparum*, and employed representative sampling methods are depicted. Prevalence points (round dots) and reports of local clinical cases (crosses) are coloured according to the proportion of infections due to *P. vivax*. Here, points are sized according to the total number of individuals examined at each site.
(TIF)

S7 Fig. Published occurrences of *P. vivax* infections in relation to the prevalence of Duffy-negativity in Africa. Dark yellow triangles indicate *P. vivax* infections in individuals with a confirmed Duffy-negative phenotype, with labels indicating national totals. Reports of any *P. vivax* infection in an area where the Duffy-negative phenotype is near fixation (frequency ≥0.95) are shown as light yellow triangles. The underlying map represents a modelled frequency map of the Duffy-negative phenotype across Africa (see [34]). Grey shading identifies areas within Africa where environmental conditions are unsuitable for *P. vivax* transmission [22].
(TIF)

S1 Table. Reports of local clinical *Plasmodium vivax* cases. NR: not reported. Rows were included in the evidence weighting if they reported at least one *P. vivax* infection and could be approximately geopositioned to a point location. Rows were included in the proportion analysis if the study design appeared to be unbiased to a particular parasite species, and could be assumed to be representative of the relative burdens of *P. vivax* and *P. falciparum* at that location.
(XLSX)

S2 Table. Reports of *Plasmodium vivax* infections detected through community prevalence surveys. NR: not reported. Rows were included in the evidence weighting if they reported at least one *P. vivax* infection and could be approximately geopositioned to a point location. Rows were included in the proportion analysis if the study design appeared to be unbiased to a particular parasite species, and could be assumed to be representative of the relative burdens of *P. vivax* and *P. falciparum* at that location.
(XLSX)

S3 Table. Reports of *Plasmodium vivax* infected *Anopheles* vectors. NR: not reported.
(XLSX)

S4 Table. Reports of *Plasmodium vivax* exposure through serological surveys. NR: not reported.
(XLSX)

S5 Table. Reports of *Plasmodium vivax* infections among Duffy-negative individuals. NR: not reported.
(XLSX)

Acknowledgments

We would like to acknowledge the following individuals and organizations for their assistance with and contribution to the imported traveller database: the European Centre for Disease Prevention and Control (ECDC) data from The European Surveillance System-TESSy and Encarna Gimenez for administrative support; the Australian Government Department of Health National Notifiable Disease Surveillance System (NNDSS) data and Rachael Corvisy for administrative support; the Centers for Disease Control and Prevention National Malaria Surveillance System (NMSS) data and Kimberly Mace for administrative support; Tim Wood, ESR New Zealand; Henrik Vedel Nielsen, Statens Serum Institut, Denmark; Gaëtan Muyldermans, Sciensano, Brussels, Belgium; Elsie Ydring, Public Health Agency of Sweden. We would also like to thank Emma Collins, Colin Johnston and Suzanne Keddie for proof-reading and Jen Rozier for GIS support.

The views and opinions of the authors expressed herein do not necessarily state or reflect those of ECDC. The accuracy of the authors' statistical analysis and the findings they report are not the responsibility of ECDC. ECDC is not responsible for conclusions or opinions drawn from the data provided. ECDC is not responsible for the correctness of the data and for data management, data merging and data collation after provision of the data. ECDC shall not be held liable for improper or incorrect use of the data.

Author Contributions

Conceptualization: Katherine E. Battle, Rosalind E. Howes.

Investigation: Katherine A. Twohig, Daniel A. Pfeffer, Katherine E. Battle, Rosalind E. Howes.

Visualization: Katherine A. Twohig, Daniel A. Pfeffer.

Writing – original draft: Katherine A. Twohig, Daniel A. Pfeffer.

Writing – review & editing: Katherine A. Twohig, Daniel A. Pfeffer, J. Kevin Baird, Ric N. Price, Peter A. Zimmerman, Simon I. Hay, Peter W. Gething, Katherine E. Battle, Rosalind E. Howes.

References

1. Cibulskis RE, Alonso P, Aponte J, Aregawi M, Barrette A, Bergeron L, et al. Malaria: Global progress 2000–2015 and future challenges. Infect Dis Poverty. 2016; 5:61. https://doi.org/10.1186/s40249-016-0151-8 PMC4901420. PMID: 27282148
2. Bhatt S, Weiss DJ, Cameron E, Bisanzio D, Mappin B, Dalrymple U, et al. The effect of malaria control on *Plasmodium falciparum* in Africa between 2000 and 2015. Nature. 2015; 526:207. https://doi.org/10.1038/nature15535 https://www.nature.com/articles/nature15535#supplementary-information. PMID: 26375008
3. Cohen JM, Smith DL, Cotter C, Ward A, Yamey G, Sabot OJ, et al. Malaria resurgence: a systematic review and assessment of its causes. Malar J. 2012; 11(1):122. https://doi.org/10.1186/1475-2875-11-122 PMID: 22531245
4. Nájera JA, González-Silva M, Alonso PL. Some lessons for the future from the Global Malaria Eradication Programme (1955–1969). PLoS Med. 2011; 8(1):e1000412. https://doi.org/10.1371/journal.pmed.1000412 PMID: 21311585
5. WHO. World Malaria Report 2017. Geneva, Switzerland: World Health Organization, 2017.
6. Lover AA, Baird JK, Gosling R, Price RN. Malaria elimination: time to target all species. Am J Trop Med Hyg. 2018; 99(1):17–23. Epub 2018/05/16. https://doi.org/10.4269/ajtmh.17-0869 PMID: 29761762; PubMed Central PMCID: PMCPMC6035869.
7. Olliaro PL, Barnwell JW, Barry A, Mendis K, Mueller I, Reeder JC, et al. Implications of *Plasmodium vivax* biology for control, elimination, and research. Am J Trop Med Hyg. 2016; 95(6 Suppl):4–14. https://doi.org/10.4269/ajtmh.16-0160 PMC5201222. PMID: 27799636
8. Howes RE, Battle KE, Mendis KN, Smith DL, Cibulskis RE, Baird JK, et al. Global epidemiology of *Plasmodium vivax*. Am J Trop Med Hyg. 2016; 95(6 Suppl):15–34. https://doi.org/10.4269/ajtmh.16-0141 PMC5198891. PMID: 27402513
9. Chen I, Clarke SE, Gosling R, Hamainza B, Killeen G, Magill A, et al. "Asymptomatic" malaria: a chronic and debilitating infection that should be treated. PLoS Med. 2016; 13(1):e1001942. https://doi.org/10.1371/journal.pmed.1001942 PMID: 26783752
10. Douglas NM, Anstey NM, Buffet PA, Poespoprodjo JR, Yeo TW, White NJ, et al. The anaemia of *Plasmodium vivax* malaria. Malar J. 2012; 11:135. https://doi.org/10.1186/1475-2875-11-135 PMID: 22540175
11. Baird JK, Valecha N, Duparc S, White NJ, Price RN. Diagnosis and treatment of *Plasmodium vivax* malaria. Am J Trop Med Hyg. 2016; 95(6_Suppl):35–51. https://doi.org/10.4269/ajtmh.16-0171.
12. Miller LH, Mason SJ, Clyde DF, McGinniss MH. The resistance factor to *Plasmodium vivax* in blacks. N Engl J Med. 1976; 295(6):302–4. https://doi.org/10.1056/NEJM197608052950602 PMID: 778616
13. Gunalan K, Niangaly A, Thera MA, Doumbo OK, Miller LH. *Plasmodium vivax* infections of Duffy-negative erythrocytes: historically undetected or a recent daptation? Trends Parasitol. 2018; 34(5):420–9. https://doi.org/10.1016/j.pt.2018.02.006. PMID: 29530446
14. Howes RE, Reiner RC Jr, Battle KE, Longbottom J, Mappin B, Ordanovich D, et al. *Plasmodium vivax* transmission in Africa. PLoS Negl Trop Dis. 2015; 9(11):e0004222. https://doi.org/10.1371/journal.pntd.0004222 PMID: 26587988
15. Zimmerman PA. *Plasmodium vivax* Infection in Duffy-negative people in Africa. Am J Trop Med Hyg. 2017; 97(3):636–8. https://doi.org/10.4269/ajtmh.17-0461. PMID: 28990906
16. Guerra CA, Hay SI, Lucioparedes LS, Gikandi PW, Tatem AJ, Noor AM, et al. Assembling a global database of malaria parasite prevalence for the Malaria Atlas Project. Malar J. 2007; 6:17. Epub 2007/02/20. https://doi.org/10.1186/1475-2875-6-17 PMID: 17306022; PubMed Central PMCID: PMCPMC1805762.
17. Moyes CL, Temperley WH, Henry AJ, Burgert CR, Hay SI. Providing open access data online to advance malaria research and control. Malar J. 2013; 12:161. Epub 2013/05/18. https://doi.org/10.1186/1475-2875-12-161 PMID: 23680401; PubMed Central PMCID: PMCPMC3662599.
18. Pfeffer DA, Lucas TCD, May D, Harris J, Rozier J, Twohig KA, et al. malariaAtlas: an R interface to global malariometric data hosted by the Malaria Atlas Project. Malar J. 2018; 17(1):352. https://doi.org/10.1186/s12936-018-2500-5 PMID: 30290815; PubMed Central PMCID: PMCPMC6173876.
19. Wickham H. tidyverse: Easily install and load the 'tidyverse' 2017 [09 Aug 2018]. R package version 1.2.1:[Available from: https://CRAN.R-project.org/package=tidyverse.
20. Bivand R, Keitt T, Rowlingson B. rgdal: Bindings for the 'Geospatial' data abstraction library. R package 2017 [09 Aug 2018]. version 1.2–16:[Available from: https://CRAN.R-project.org/package=rgdal.
21. Bivand R, Pebesma E, Gomez-Rubio V. Applied spatial data analysis with R. Second edition ed. New York: Springer; 2013.

22. Guerra CA, Howes RE, Patil AP, Gething PW, Van Boeckel TP, Temperley WH, et al. The international limits and population at risk of *Plasmodium vivax* transmission in 2009. PLoS Negl Trop Dis. 2010; 4(8): e774. Epub 2010/08/07. https://doi.org/10.1371/journal.pntd.0000774 PMID: 20689816; PubMed Central PMCID: PMCPMC2914753.

23. Global Administrative Areas: GADM database of Global Administrative Areas; 2018. 2.8:[Available from: www.gadm.org.

24. Aragon TJ. epitools: Epidemiology tools 2017 [09 Aug 2018]. R package version 0.5–10:[Available from: https://CRAN.R-project.org/package=epitools.

25. Cao J, Sturrock HJ, Cotter C, Zhou S, Zhou H, Liu Y, et al. Communicating and monitoring surveillance and response activities for malaria elimination: China's "1-3-7" strategy. PLoS Med. 2014; 11(5): e1001642. Epub 2014/05/16. https://doi.org/10.1371/journal.pmed.1001642 PMID: 24824170; PubMed Central PMCID: PMCPMC4019513.

26. Jovani R, Tella JL. Parasite prevalence and sample size: misconceptions and solutions. Trends Parasitol. 2006; 22(5):214–8. https://doi.org/10.1016/j.pt.2006.02.011 PMID: 16531119

27. The DHS Program. Demographic and Health Surveys 2018 [Apr 2018]. Available from: https://dhsprogram.com/.

28. Malariasurveys.org. Malaria Indicator Surveys 2018 [Apr 2018]. Available from: http://www.malariasurveys.org/.

29. Lekweiry KM, Salem MS, Basco LK, Briolant S, Hafid J, Boukhary AO. Malaria in Mauritania: retrospective and prospective overview. Malar J. 2015; 14:100. Epub 2015/04/17. https://doi.org/10.1186/s12936-015-0607-5 PMID: 25880759; PubMed Central PMCID: PMCPMC4350613.

30. Cook J, Xu W, Msellem M, Vonk M, Bergstrom B, Gosling R, et al. Mass screening and treatment on the basis of results of a *Plasmodium falciparum*-specific rapid diagnostic test did not reduce malaria incidence in Zanzibar. J Infect Dis. 2015; 211(9):1476–83. Epub 2014/11/28. https://doi.org/10.1093/infdis/jiu655 PMID: 25429102.

31. Collins WE, Jeffery GM. *Plasmodium ovale*: parasite and disease. Clin Microbiol Rev. 2005; 18(3):570–81. Epub 2005/07/16. https://doi.org/10.1128/CMR.18.3.570-581.2005 PMID: 16020691; PubMed Central PMCID: PMC1195966.

32. Prugnolle F, Rougeron V, Becquart P, Berry A, Makanga B, Rahola N, et al. Diversity, host switching and evolution of *Plasmodium vivax* infecting African great apes. Proc Natl Acad Sci U S A. 2013; 110 (20):8123–8. Epub 2013/05/03. https://doi.org/10.1073/pnas.1306004110 [pii]. PMID: 23637341; PubMed Central PMCID: PMC3657773.

33. Battle KE, Karhunen MS, Bhatt S, Gething PW, Howes RE, Golding N, et al. Geographical variation in *Plasmodium vivax* relapse. Malar J. 2014; 13:144. https://doi.org/10.1186/1475-2875-13-144 PMID: 24731298; PubMed Central PMCID: PMCPMC4021508.

34. Gething PW, Elyazar IR, Moyes CL, Smith DL, Battle KE, Guerra CA, et al. A long neglected world malaria map: *Plasmodium vivax* endemicity in 2010. PLoS Negl Trop Dis. 2012; 6(9):e1814. Epub 2012/09/13. https://doi.org/10.1371/journal.pntd.0001814 PMID: 22970336; PubMed Central PMCID: PMCPMC3435256.

35. Howes RE, Patil AP, Piel FB, Nyangiri OA, Kabaria CW, Gething PW, et al. The global distribution of the Duffy blood group. Nat Commun. 2011; 2:266. https://doi.org/10.1038/ncomms1265 https://www.nature.com/articles/ncomms1265#supplementary-information. PMID: 21468018

36. Cotter C, Sturrock HJ, Hsiang MS, Liu J, Phillips AA, Hwang J, et al. The changing epidemiology of malaria elimination: new strategies for new challenges. Lancet. 2013; 382(9895):900–11. Epub 2013/04/19. https://doi.org/10.1016/S0140-6736(13)60310-4 PMID: 23594387.

37. Gunalan K, Lo E, Hostetler JB, Yewhalaw D, Mu J, Neafsey DE, et al. Role of *Plasmodium vivax* Duffy-binding protein 1 in invasion of Duffy-null Africans. Proc Natl Acad Sci U S A. 2016; 113(22):6271–6. Epub 2016/05/18. https://doi.org/10.1073/pnas.1606113113 PMID: 27190089; PubMed Central PMCID: PMCPMC4896682.

38. Gruszczyk J, Kanjee U, Chan LJ, Menant S, Malleret B, Lim NTY, et al. Transferrin receptor 1 is a reticulocyte-specific receptor for *Plasmodium vivax*. Science. 2018; 359(6371):48–55. Epub 2018/01/06. https://doi.org/10.1126/science.aan1078 PMID: 29302006; PubMed Central PMCID: PMCPMC5788258.

39. Malleret BES, Abbas; Howland, Shanshan Wu; Suwanarusk, Rossarin; Ong, Alice S. M.; Kosaisavee, Varakorn; Chu, Trang T.T.; Sinha, Ameya; Gruszczyk, Jakob; Collin, Yves; Bertrand, Oliver; Lescar, Julien; Maurer-Stroh, Sebastian; Snounou, Georges; Tham, Wai-Hong; Chandramohanadas, Rajesh; Nosten, Francois; Russell, Bruce; Renia, Laurent, editor CD98 is a Plasmodium vivax receptor for human reticulocytes. Proceedings of International Conference on Plasmodium vivax; 2018; Campinas, Brazil: Galoá.

40. Chihanga S, Haque U, Chanda E, Mosweunyane T, Moakofhi K, Jibril HB, et al. Malaria elimination in Botswana, 2012–2014: achievements and challenges. Parasit Vectors. 2016; 9(1):99. https://doi.org/10.1186/s13071-016-1382-z PMID: 26911433

41. Moakofhi K, Edwards JK, Motlaleng M, Namboze J, Butt W, Obopile M, et al. Advances in malaria elimination in Botswana: a dramatic shift to parasitological diagnosis, 2008–2014. Public Health Action. 2018; 8(Suppl 1):S34–S8. https://doi.org/10.5588/pha.17.0017 PMC5912420. PMID: 29713592

42. Motlaleng M, Edwards J, Namboze J, Butt W, Moakofhi K, Obopile M, et al. Driving towards malaria elimination in Botswana by 2018: progress on case-based surveillance, 2013–2014. Public Health Action. 2018; 8(Suppl 1):S24–S8. https://doi.org/10.5588/pha.17.0019 PMC5912417. PMID: 29713590

43. Motshoge T, Ababio GK, Aleksenko L, Read J, Peloewetse E, Loeto M, et al. Molecular evidence of high rates of asymptomatic *P. vivax* infection and very low *P. falciparum* malaria in Botswana. BMC Infect Dis. 2016; 16:520. Epub 2016/09/30. https://doi.org/10.1186/s12879-016-1857-8 PMID: 27682611; PubMed Central PMCID: PMCPMC5041318.

44. Motshoge T, Ababio G, Aleksenko L, Souda S, Muthoga CW, Mutukwa N, et al. Prevalence of G6PD deficiency and associated haematological parameters in children from Botswana. Infect Genet Evol. 2018; 63:73–8. Epub 2018/05/21. https://doi.org/10.1016/j.meegid.2018.05.014 PMID: 29778768.

45. Barbosa S, Gozze AB, Lima NF, Batista CL, Bastos Mda S, Nicolete VC, et al. Epidemiology of disappearing *Plasmodium vivax* malaria: a case study in rural Amazonia. PLoS Negl Trop Dis. 2014; 8(8): e3109. Epub 2014/08/29. https://doi.org/10.1371/journal.pntd.0003109 PMID: 25166263; PubMed Central PMCID: PMCPMC4148206.

46. Lin JT, Saunders DL, Meshnick SR. The role of submicroscopic malaria in malaria transmission: what is the evidence? Trends Parasitol. 2014; 30(4):183–90. https://doi.org/10.1016/j.pt.2014.02.004 PMC4049069. PMID: 24642035

47. Open Society Initiative for Southern Africa (OSISA), Oxfam. Strengthening popular participation in the African Union: a guide to AU structures and processes. 2009.
